# Benzyl 2,5-dioxopyrrolidin-1-yl carbonate

**DOI:** 10.1107/S1600536808015948

**Published:** 2008-06-21

**Authors:** An-Fu Hu, Tao Ji, Yu-Xing Gao, Peng-Xiang Xu, Yu-Fen Zhao

**Affiliations:** a Department of Chemistry, The Key Laboratory for Chemical Biology of Fujian Province, College of Chemistry and Chemical Engineering, Xiamen University, Xiamen 361005, People’s Republic of China

## Abstract

The asymmetric unit of the title compound, C_12_H_11_NO_5_, contains two independent mol­ecules with similar geometric parameters but different orientations of the phenyl rings. The mol­ecular packing is stabilized by weak nonclassical C—H⋯O hydrogen-bonding inter­actions.

## Related literature

For related literature, see: Alenka (1982 ▶); Wang *et al.* (2006 ▶).
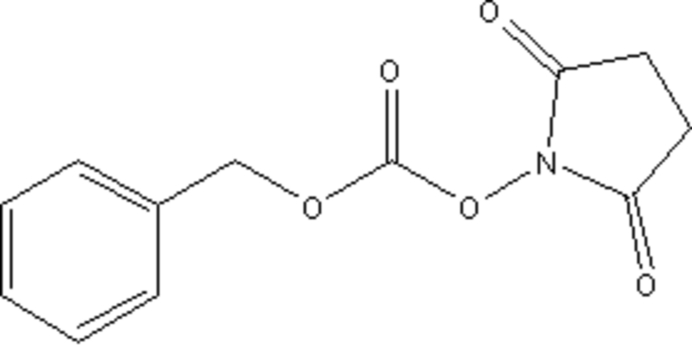


## Experimental

### 

#### Crystal data

C_12_H_11_NO_5_
*M* *_r_* = 249.22
Monoclinic, 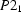

*a* = 12.9348 (6) Å
*b* = 6.0151 (3) Å
*c* = 16.5398 (9) Å
β = 106.170 (5)°
*V* = 1235.96 (11) Å^3^
*Z* = 4
Mo *K*α radiation
μ = 0.11 mm^−1^
*T* = 293 (2) K
0.60 × 0.50 × 0.37 mm

#### Data collection

Bruker APEX area-detector diffractometer
Absorption correction: multi-scan (*SADABS*; Bruker, 2001 ▶) *T*_min_ = 0.939, *T*_max_ = 0.962
7601 measured reflections
2648 independent reflections
1646 reflections with *I* > 2σ(*I*)
*R*_int_ = 0.029

#### Refinement

*R*[*F* ^2^ > 2σ(*F* ^2^)] = 0.037
*wR*(*F* ^2^) = 0.113
*S* = 0.90
2648 reflections
325 parameters
1 restraint
H-atom parameters constrained
Δρ_max_ = 0.16 e Å^−3^
Δρ_min_ = −0.16 e Å^−3^

### 

Data collection: *SMART* (Bruker, 2001 ▶); cell refinement: *SAINT* (Bruker, 2001 ▶); data reduction: *SAINT*; program(s) used to solve structure: *SHELXS97* (Sheldrick, 2008 ▶); program(s) used to refine structure: *SHELXL97* (Sheldrick, 2008 ▶); molecular graphics: *ORTEP-3 for Windows* (Farrugia, 1997 ▶); software used to prepare material for publication: *SHELXL97*.

## Supplementary Material

Crystal structure: contains datablocks I, global. DOI: 10.1107/S1600536808015948/pv2076sup1.cif

Structure factors: contains datablocks I. DOI: 10.1107/S1600536808015948/pv2076Isup2.hkl

Additional supplementary materials:  crystallographic information; 3D view; checkCIF report

## Figures and Tables

**Table 1:** Hydrogen-bond geometry (Å, °)

*D*—H⋯*A*	*D*—H	H⋯*A*	*D*⋯*A*	*D*—H⋯*A*
C10—H10*B*⋯O1^i^	0.97	2.56	3.251 (4)	128
C8*A*—H8*AA*⋯O4^ii^	0.93	2.47	3.398 (5)	172
C10—H10*A*⋯O1*A*^ii^	0.97	2.48	3.057 (5)	118
C2*A*—H2*AA*⋯O1^iii^	0.97	2.49	3.417 (5)	161
C6*A*—H6*AA*⋯O5*A*^iv^	0.93	2.60	3.354 (6)	139

Symmetry codes: (i) 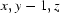; (ii) 
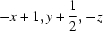
; (iii) 
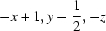
; (iv) 
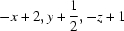
.

**Fig. 1.**
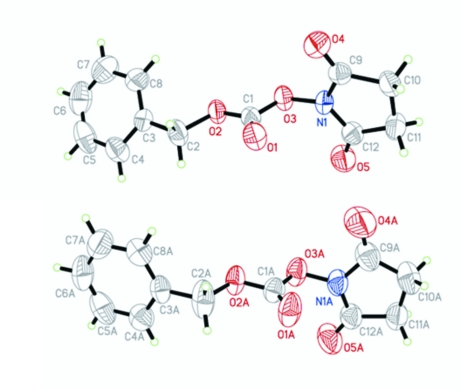
The two independent molecules of (I) in the asymmetric unit, showing the atom-numbering scheme. Displacement ellipsoids are drawn at the 50% probability level (arbitrary spheres for H atoms).

## supplementary crystallographic information

### Comment

The title compound, (I), is a more convenient and mild reagent than benzyl carbonochloridate in protecting amino acids. It can also be used in the synthesis of a series of biologically active molecules (Alenka, 1982).

The asymmetric unit of (I) contains two independent molecules, the atoms of the second molecule have been identified by the letter A in their labels (Fig. 1). The two molecules adopt different orientations of the phenyl rings, as reflected by the torsion angles O2—C2—C3—C8 and O2—C2—C3—C4 with values -48.6 (4) and 134.7 (3)°, respectively, in the first molecule as compared with the values of the corresponding torsion angles in the second molecule being -88.5 (4) and 95.3 (5)°, respectively. Furthermore, the bond lengths and angles also have some slight differences. For example, the bond lengths O3—N1 and O3A—N1A are 1.391 (3) and 1.376 (4) Å, respectively. As to the bond angles, the values of the angles O1—C1—O3 and O1A—C1A—O3A are 124.9 (3) and 123.3 (3)°, respectively. Bond lengths and angles in (I) are in agreement with those reported for a similar compound (Wang *et al.*, 2006). The structure contains rather weak non-classical hydrogen bonds of the type C—H···O involving the carbonyl groups (Table 1).

### Experimental

To a stirred solution of benzyl chloroformate (3.41 g, 20 mmol) and *N*-hydroxysuccinimide (2.30 g, 20 mmol) in methylene chloride (20 ml) at room temperature was added dropwise triethylamine (2.90 ml, 20 mmol). After stirring for 10 h at room temperature, the mixture was concentrated under vacuum and the crude product was purified by column chromatography (petroleum ether-ethyl acetate, 4:1) to give the title compound as a white solid in 88% yield. Single crystals of (I) were obtained by slow evaporation of a petroleum ether-ethyl acetate solution (1:1 *v*/*v*).

### Refinement

All H atoms were placed in geometrically idealized positions and constrained to ride on their parent atoms, with C—H = 0.93 Å (aromatic) and 0.97 Å (methylene), with *U*_iso_(H) = 1.2*U*_eq_(C) for all H atoms. In the absence of significant anomalous scattering effects, the absolute configuration of (I) could not be determined. Therefore, Friedel pairs (1632) were merged.

### Crystal data

**Table:**

C_12_H_11_NO_5_	*F*_000_ = 520
*M**_r_* = 249.22	*D*_x_ = 1.339 Mg m^−^^3^
Monoclinic, *P*2_1_	Mo *K*α radiation λ = 0.71073 Å
Hall symbol: P 2yb	Cell parameters from 2829 reflections
*a* = 12.9348 (6) Å	θ = 2.6–32.7º
*b* = 6.0151 (3) Å	µ = 0.11 mm^−^^1^
*c* = 16.5398 (9) Å	*T* = 293 (2) K
β = 106.170 (5)º	Block, colorless
*V* = 1235.96 (11) Å^3^	0.60 × 0.50 × 0.37 mm
*Z* = 4	

### Data collection

**Table:**

Bruker APEX area-detector diffractometer	2648 independent reflections
Radiation source: fine-focus sealed tube	1646 reflections with *I* > 2σ(*I*)
Monochromator: graphite	*R*_int_ = 0.029
*T* = 293(2) K	θ_max_ = 26.0º
φ and ω scans	θ_min_ = 2.6º
Absorption correction: Multi-scan(SADABS; Bruker, 2001)	*h* = −15→15
*T*_min_ = 0.939, *T*_max_ = 0.962	*k* = −7→6
7601 measured reflections	*l* = −20→20

### Refinement

**Table:**

Refinement on *F*^2^	Secondary atom site location: difference Fourier map
Least-squares matrix: full	Hydrogen site location: inferred from neighbouring sites
*R*[*F*^2^ > 2σ(*F*^2^)] = 0.037	H-atom parameters constrained
*wR*(*F*^2^) = 0.113	*w* = 1/[σ^2^(*F*_o_^2^) + (0.0784*P*)^2^] where *P* = (*F*_o_^2^ + 2*F*_c_^2^)/3
*S* = 0.90	(Δ/σ)_max_ < 0.001
2648 reflections	Δρ_max_ = 0.16 e Å^−^^3^
325 parameters	Δρ_min_ = −0.16 e Å^−^^3^
1 restraint	Extinction correction: none
Primary atom site location: structure-invariant direct methods	

### Special details

**Table:**

Geometry. All e.s.d.'s (except the e.s.d. in the dihedral angle between two l.s. planes) are estimated using the full covariance matrix. The cell e.s.d.'s are taken into account individually in the estimation of e.s.d.'s in distances, angles and torsion angles; correlations between e.s.d.'s in cell parameters are only used when they are defined by crystal symmetry. An approximate (isotropic) treatment of cell e.s.d.'s is used for estimating e.s.d.'s involving l.s. planes.
Refinement. Refinement of *F*^2^ against ALL reflections. The weighted *R*-factor *wR* and goodness of fit *S* are based on *F*^2^, conventional *R*-factors *R* are based on *F*, with *F* set to zero for negative *F*^2^. The threshold expression of *F*^2^ > σ(*F*^2^) is used only for calculating *R*-factors(gt) *etc*. and is not relevant to the choice of reflections for refinement. *R*-factors based on *F*^2^ are statistically about twice as large as those based on *F*, and *R*- factors based on ALL data will be even larger.

### Fractional atomic coordinates and isotropic or equivalent isotropic displacement parameters (Å^2^)

**Table:**

	*x*	*y*	*z*	*U*_iso_*/*U*_eq_	
O1	0.27634 (19)	0.9193 (4)	−0.12266 (14)	0.0590 (6)	
O2	0.23745 (17)	0.8957 (3)	0.00217 (13)	0.0497 (5)	
O3	0.31938 (17)	0.6230 (4)	−0.03469 (13)	0.0505 (6)	
O4	0.21313 (18)	0.4121 (5)	−0.18601 (15)	0.0656 (6)	
O5	0.53866 (19)	0.6359 (5)	−0.02062 (18)	0.0748 (8)	
N1	0.36798 (19)	0.5329 (4)	−0.09240 (16)	0.0455 (6)	
C1	0.2760 (2)	0.8299 (5)	−0.0593 (2)	0.0444 (7)	
C2	0.1931 (3)	1.1212 (6)	−0.0092 (2)	0.0608 (9)	
H2A	0.1403	1.1337	−0.0637	0.073*	
H2B	0.2500	1.2278	−0.0070	0.073*	
C3	0.1417 (2)	1.1683 (6)	0.05872 (19)	0.0472 (8)	
C4	0.1608 (3)	1.3686 (7)	0.1003 (2)	0.0679 (10)	
H4A	0.2106	1.4682	0.0900	0.081*	
C5	0.1038 (4)	1.4196 (9)	0.1584 (3)	0.0840 (13)	
H5A	0.1137	1.5575	0.1849	0.101*	
C6	0.0353 (4)	1.2742 (11)	0.1766 (3)	0.0891 (15)	
H6A	0.0001	1.3084	0.2170	0.107*	
C7	0.0174 (4)	1.0773 (10)	0.1359 (3)	0.0933 (15)	
H7A	−0.0321	0.9779	0.1467	0.112*	
C8	0.0722 (3)	1.0237 (8)	0.0785 (2)	0.0661 (10)	
H8A	0.0613	0.8850	0.0526	0.079*	
C9	0.3091 (2)	0.4209 (5)	−0.16335 (19)	0.0461 (7)	
C10	0.3907 (3)	0.3163 (6)	−0.1998 (2)	0.0565 (9)	
H10A	0.3761	0.3525	−0.2591	0.068*	
H10B	0.3903	0.1560	−0.1938	0.068*	
C11	0.4985 (3)	0.4128 (7)	−0.1500 (2)	0.0623 (9)	
H11A	0.5500	0.2946	−0.1289	0.075*	
H11B	0.5271	0.5101	−0.1853	0.075*	
C12	0.4779 (3)	0.5406 (6)	−0.0785 (2)	0.0553 (9)	
O1A	0.6443 (2)	0.2514 (6)	0.29926 (19)	0.0827 (9)	
O2A	0.77413 (16)	0.4716 (5)	0.37734 (14)	0.0616 (7)	
O3A	0.62034 (17)	0.4715 (5)	0.40198 (16)	0.0689 (7)	
O4A	0.4335 (3)	0.6375 (7)	0.2938 (3)	0.1198 (14)	
O5A	0.5736 (2)	0.0935 (7)	0.4792 (2)	0.1059 (12)	
N1A	0.5208 (2)	0.3728 (6)	0.3867 (2)	0.0622 (8)	
C1A	0.6797 (3)	0.3790 (7)	0.3534 (2)	0.0550 (8)	
C2A	0.8508 (3)	0.3870 (10)	0.3332 (3)	0.0911 (16)	
H2AA	0.8264	0.4263	0.2740	0.109*	
H2AB	0.8552	0.2262	0.3375	0.109*	
C3A	0.9578 (2)	0.4857 (7)	0.3717 (2)	0.0557 (9)	
C4A	1.0340 (3)	0.3744 (8)	0.4333 (2)	0.0709 (11)	
H4AA	1.0177	0.2372	0.4527	0.085*	
C5A	1.1365 (3)	0.4690 (11)	0.4670 (3)	0.0887 (16)	
H5AA	1.1874	0.3968	0.5099	0.106*	
C6A	1.1608 (3)	0.6646 (11)	0.4368 (3)	0.0895 (15)	
H6AA	1.2292	0.7252	0.4578	0.107*	
C7A	1.0853 (4)	0.7737 (9)	0.3755 (3)	0.0862 (13)	
H7AA	1.1020	0.9096	0.3555	0.103*	
C8A	0.9857 (3)	0.6845 (8)	0.3435 (3)	0.0706 (11)	
H8AA	0.9353	0.7605	0.3015	0.085*	
C9A	0.4312 (3)	0.4689 (9)	0.3325 (3)	0.0754 (12)	
C10A	0.3393 (3)	0.3194 (10)	0.3337 (3)	0.0832 (14)	
H10C	0.3091	0.2524	0.2788	0.100*	
H10D	0.2831	0.4018	0.3490	0.100*	
C11A	0.3865 (3)	0.1416 (8)	0.3994 (3)	0.0777 (12)	
H11C	0.3544	0.1502	0.4458	0.093*	
H11D	0.3736	−0.0056	0.3747	0.093*	
C12A	0.5045 (3)	0.1884 (8)	0.4293 (3)	0.0673 (11)	

### Atomic displacement parameters (Å^2^)

**Table:**

	*U*^11^	*U*^22^	*U*^33^	*U*^12^	*U*^13^	*U*^23^
O1	0.0811 (15)	0.0513 (14)	0.0528 (13)	0.0115 (13)	0.0323 (12)	0.0095 (12)
O2	0.0626 (12)	0.0399 (12)	0.0566 (12)	0.0124 (11)	0.0329 (10)	0.0057 (11)
O3	0.0624 (13)	0.0440 (13)	0.0512 (13)	0.0133 (11)	0.0258 (10)	0.0033 (10)
O4	0.0546 (14)	0.0698 (16)	0.0720 (15)	−0.0004 (13)	0.0168 (11)	−0.0030 (14)
O5	0.0559 (14)	0.0745 (18)	0.0844 (18)	0.0002 (14)	0.0039 (12)	−0.0231 (17)
N1	0.0468 (14)	0.0476 (16)	0.0459 (14)	0.0087 (12)	0.0194 (12)	−0.0030 (13)
C1	0.0493 (17)	0.0382 (18)	0.0488 (18)	0.0030 (14)	0.0188 (14)	0.0032 (15)
C2	0.080 (2)	0.043 (2)	0.068 (2)	0.0177 (18)	0.0362 (19)	0.0118 (18)
C3	0.0414 (15)	0.051 (2)	0.0494 (18)	0.0076 (16)	0.0135 (13)	−0.0005 (16)
C4	0.088 (2)	0.053 (2)	0.066 (2)	0.003 (2)	0.0270 (19)	−0.005 (2)
C5	0.118 (3)	0.067 (3)	0.065 (2)	0.027 (3)	0.022 (2)	−0.013 (2)
C6	0.098 (3)	0.110 (4)	0.072 (3)	0.032 (3)	0.043 (3)	0.001 (3)
C7	0.092 (3)	0.101 (4)	0.109 (3)	−0.004 (3)	0.064 (3)	0.000 (3)
C8	0.064 (2)	0.064 (2)	0.076 (3)	−0.0068 (19)	0.0300 (19)	−0.008 (2)
C9	0.0498 (18)	0.0416 (17)	0.0467 (17)	0.0004 (16)	0.0131 (14)	0.0059 (16)
C10	0.077 (2)	0.0432 (19)	0.056 (2)	0.0037 (17)	0.0298 (17)	−0.0018 (16)
C11	0.0611 (19)	0.062 (2)	0.071 (2)	0.010 (2)	0.0308 (17)	−0.004 (2)
C12	0.057 (2)	0.045 (2)	0.066 (2)	0.0055 (17)	0.0220 (18)	0.0045 (18)
O1A	0.0602 (15)	0.104 (2)	0.088 (2)	−0.0266 (16)	0.0275 (14)	−0.0459 (19)
O2A	0.0461 (12)	0.0834 (18)	0.0610 (13)	−0.0171 (12)	0.0241 (10)	−0.0254 (13)
O3A	0.0544 (13)	0.0811 (18)	0.0809 (16)	−0.0165 (13)	0.0347 (12)	−0.0239 (15)
O4A	0.100 (2)	0.129 (3)	0.137 (3)	0.019 (2)	0.045 (2)	0.071 (3)
O5A	0.0670 (17)	0.125 (3)	0.121 (3)	0.0130 (19)	0.0171 (17)	0.058 (2)
N1A	0.0476 (16)	0.077 (2)	0.0663 (17)	−0.0042 (16)	0.0236 (14)	0.0057 (17)
C1A	0.0526 (19)	0.063 (2)	0.0530 (18)	−0.0064 (18)	0.0210 (15)	−0.004 (2)
C2A	0.064 (2)	0.137 (4)	0.087 (3)	−0.024 (3)	0.044 (2)	−0.057 (3)
C3A	0.0461 (17)	0.080 (3)	0.0450 (18)	−0.0014 (19)	0.0186 (15)	−0.0142 (19)
C4A	0.073 (3)	0.081 (3)	0.066 (2)	0.005 (2)	0.031 (2)	0.006 (2)
C5A	0.061 (2)	0.136 (5)	0.064 (2)	0.028 (3)	0.0094 (19)	0.004 (3)
C6A	0.051 (2)	0.129 (5)	0.086 (3)	−0.019 (3)	0.015 (2)	−0.024 (4)
C7A	0.084 (3)	0.081 (3)	0.101 (3)	−0.018 (3)	0.039 (3)	−0.012 (3)
C8A	0.065 (2)	0.082 (3)	0.065 (2)	0.010 (2)	0.0181 (18)	0.001 (2)
C9A	0.063 (2)	0.099 (4)	0.067 (2)	0.008 (2)	0.0224 (19)	0.014 (3)
C10A	0.056 (2)	0.123 (4)	0.072 (3)	0.004 (3)	0.0203 (18)	0.014 (3)
C11A	0.056 (2)	0.085 (3)	0.091 (3)	−0.010 (2)	0.0193 (18)	0.006 (3)
C12A	0.056 (2)	0.077 (3)	0.073 (3)	0.000 (2)	0.0241 (19)	0.010 (2)

### Geometric parameters (Å, °)

**Table:**

O1—C1	1.179 (4)	O1A—C1A	1.171 (4)
O2—C1	1.312 (4)	O2A—C1A	1.300 (4)
O2—C2	1.464 (4)	O2A—C2A	1.477 (4)
O3—C1	1.379 (4)	O3A—C1A	1.374 (4)
O3—N1	1.391 (3)	O3A—N1A	1.376 (4)
O4—C9	1.193 (4)	O4A—C9A	1.205 (6)
O5—C12	1.202 (4)	O5A—C12A	1.182 (5)
N1—C12	1.376 (4)	N1A—C12A	1.361 (5)
N1—C9	1.383 (4)	N1A—C9A	1.379 (5)
C2—C3	1.484 (5)	C2A—C3A	1.477 (5)
C2—H2A	0.9700	C2A—H2AA	0.9700
C2—H2B	0.9700	C2A—H2AB	0.9700
C3—C8	1.355 (5)	C3A—C8A	1.368 (6)
C3—C4	1.375 (5)	C3A—C4A	1.378 (5)
C4—C5	1.399 (6)	C4A—C5A	1.407 (6)
C4—H4A	0.9300	C4A—H4AA	0.9300
C5—C6	1.338 (7)	C5A—C6A	1.349 (8)
C5—H5A	0.9300	C5A—H5AA	0.9300
C6—C7	1.350 (8)	C6A—C7A	1.364 (7)
C6—H6A	0.9300	C6A—H6AA	0.9300
C7—C8	1.371 (6)	C7A—C8A	1.359 (6)
C7—H7A	0.9300	C7A—H7AA	0.9300
C8—H8A	0.9300	C8A—H8AA	0.9300
C9—C10	1.493 (5)	C9A—C10A	1.495 (6)
C10—C11	1.524 (5)	C10A—C11A	1.526 (7)
C10—H10A	0.9700	C10A—H10C	0.9700
C10—H10B	0.9700	C10A—H10D	0.9700
C11—C12	1.495 (5)	C11A—C12A	1.493 (6)
C11—H11A	0.9700	C11A—H11C	0.9700
C11—H11B	0.9700	C11A—H11D	0.9700
			
C1—O2—C2	113.6 (2)	C1A—O2A—C2A	113.9 (3)
C1—O3—N1	112.0 (2)	C1A—O3A—N1A	111.3 (3)
C12—N1—C9	117.2 (3)	C12A—N1A—O3A	121.9 (3)
C12—N1—O3	121.0 (3)	C12A—N1A—C9A	117.0 (3)
C9—N1—O3	121.6 (2)	O3A—N1A—C9A	120.8 (3)
O1—C1—O2	130.4 (3)	O1A—C1A—O2A	130.4 (3)
O1—C1—O3	124.9 (3)	O1A—C1A—O3A	123.3 (3)
O2—C1—O3	104.8 (3)	O2A—C1A—O3A	106.2 (3)
O2—C2—C3	108.8 (3)	O2A—C2A—C3A	109.0 (3)
O2—C2—H2A	109.9	O2A—C2A—H2AA	109.9
C3—C2—H2A	109.9	C3A—C2A—H2AA	109.9
O2—C2—H2B	109.9	O2A—C2A—H2AB	109.9
C3—C2—H2B	109.9	C3A—C2A—H2AB	109.9
H2A—C2—H2B	108.3	H2AA—C2A—H2AB	108.3
C8—C3—C4	118.7 (3)	C8A—C3A—C4A	118.3 (3)
C8—C3—C2	121.8 (3)	C8A—C3A—C2A	120.6 (4)
C4—C3—C2	119.4 (3)	C4A—C3A—C2A	121.0 (4)
C3—C4—C5	118.7 (4)	C3A—C4A—C5A	119.8 (4)
C3—C4—H4A	120.7	C3A—C4A—H4AA	120.1
C5—C4—H4A	120.7	C5A—C4A—H4AA	120.1
C6—C5—C4	121.3 (4)	C6A—C5A—C4A	119.8 (4)
C6—C5—H5A	119.3	C6A—C5A—H5AA	120.1
C4—C5—H5A	119.3	C4A—C5A—H5AA	120.1
C5—C6—C7	119.5 (4)	C5A—C6A—C7A	120.1 (4)
C5—C6—H6A	120.2	C5A—C6A—H6AA	119.9
C7—C6—H6A	120.2	C7A—C6A—H6AA	119.9
C6—C7—C8	120.2 (5)	C8A—C7A—C6A	120.3 (5)
C6—C7—H7A	119.9	C8A—C7A—H7AA	119.9
C8—C7—H7A	119.9	C6A—C7A—H7AA	119.9
C3—C8—C7	121.4 (4)	C7A—C8A—C3A	121.6 (4)
C3—C8—H8A	119.3	C7A—C8A—H8AA	119.2
C7—C8—H8A	119.3	C3A—C8A—H8AA	119.2
O4—C9—N1	124.6 (3)	O4A—C9A—N1A	123.9 (4)
O4—C9—C10	130.1 (3)	O4A—C9A—C10A	130.6 (4)
N1—C9—C10	105.3 (3)	N1A—C9A—C10A	105.5 (4)
C9—C10—C11	105.3 (3)	C9A—C10A—C11A	105.6 (3)
C9—C10—H10A	110.7	C9A—C10A—H10C	110.6
C11—C10—H10A	110.7	C11A—C10A—H10C	110.6
C9—C10—H10B	110.7	C9A—C10A—H10D	110.6
C11—C10—H10B	110.7	C11A—C10A—H10D	110.6
H10A—C10—H10B	108.8	H10C—C10A—H10D	108.8
C12—C11—C10	106.6 (3)	C12A—C11A—C10A	105.8 (4)
C12—C11—H11A	110.4	C12A—C11A—H11C	110.6
C10—C11—H11A	110.4	C10A—C11A—H11C	110.6
C12—C11—H11B	110.4	C12A—C11A—H11D	110.6
C10—C11—H11B	110.4	C10A—C11A—H11D	110.6
H11A—C11—H11B	108.6	H11C—C11A—H11D	108.7
O5—C12—N1	124.4 (3)	O5A—C12A—N1A	123.9 (4)
O5—C12—C11	130.8 (3)	O5A—C12A—C11A	130.1 (4)
N1—C12—C11	104.7 (3)	N1A—C12A—C11A	105.9 (3)
			
C1—O3—N1—C12	100.4 (3)	C1A—O3A—N1A—C12A	88.0 (4)
C1—O3—N1—C9	−83.5 (3)	C1A—O3A—N1A—C9A	−97.8 (4)
C2—O2—C1—O1	−3.5 (5)	C2A—O2A—C1A—O1A	−5.6 (6)
C2—O2—C1—O3	176.4 (2)	C2A—O2A—C1A—O3A	178.3 (4)
N1—O3—C1—O1	2.2 (4)	N1A—O3A—C1A—O1A	8.3 (5)
N1—O3—C1—O2	−177.7 (2)	N1A—O3A—C1A—O2A	−175.2 (3)
C1—O2—C2—C3	174.0 (3)	C1A—O2A—C2A—C3A	−173.9 (4)
O2—C2—C3—C8	−48.6 (4)	O2A—C2A—C3A—C8A	−88.5 (4)
O2—C2—C3—C4	134.7 (3)	O2A—C2A—C3A—C4A	95.3 (5)
C8—C3—C4—C5	−3.0 (5)	C8A—C3A—C4A—C5A	1.6 (5)
C2—C3—C4—C5	173.8 (3)	C2A—C3A—C4A—C5A	177.8 (3)
C3—C4—C5—C6	2.9 (6)	C3A—C4A—C5A—C6A	−2.2 (6)
C4—C5—C6—C7	−2.6 (7)	C4A—C5A—C6A—C7A	1.8 (7)
C5—C6—C7—C8	2.4 (7)	C5A—C6A—C7A—C8A	−1.0 (7)
C4—C3—C8—C7	3.0 (6)	C6A—C7A—C8A—C3A	0.4 (6)
C2—C3—C8—C7	−173.8 (4)	C4A—C3A—C8A—C7A	−0.7 (5)
C6—C7—C8—C3	−2.6 (7)	C2A—C3A—C8A—C7A	−177.0 (4)
C12—N1—C9—O4	−175.3 (3)	C12A—N1A—C9A—O4A	176.7 (5)
O3—N1—C9—O4	8.4 (5)	O3A—N1A—C9A—O4A	2.2 (6)
C12—N1—C9—C10	6.2 (4)	C12A—N1A—C9A—C10A	−4.1 (5)
O3—N1—C9—C10	−170.1 (3)	O3A—N1A—C9A—C10A	−178.6 (3)
O4—C9—C10—C11	172.9 (3)	O4A—C9A—C10A—C11A	−176.5 (5)
N1—C9—C10—C11	−8.8 (4)	N1A—C9A—C10A—C11A	4.4 (5)
C9—C10—C11—C12	8.6 (4)	C9A—C10A—C11A—C12A	−3.4 (5)
C9—N1—C12—O5	178.3 (3)	O3A—N1A—C12A—O5A	−3.4 (6)
O3—N1—C12—O5	−5.4 (5)	C9A—N1A—C12A—O5A	−177.8 (5)
C9—N1—C12—C11	−0.7 (4)	O3A—N1A—C12A—C11A	176.3 (4)
O3—N1—C12—C11	175.7 (3)	C9A—N1A—C12A—C11A	1.9 (5)
C10—C11—C12—O5	176.0 (4)	C10A—C11A—C12A—O5A	−179.2 (5)
C10—C11—C12—N1	−5.1 (4)	C10A—C11A—C12A—N1A	1.1 (5)

### Hydrogen-bond geometry (Å, °)

**Table:**

*D*—H···*A*	*D*—H	H···*A*	*D*···*A*	*D*—H···*A*
C10—H10B···O1^i^	0.97	2.56	3.251 (4)	128
C8A—H8AA···O4^ii^	0.93	2.47	3.398 (5)	172
C10—H10A···O1A^ii^	0.97	2.48	3.057 (5)	118
C2A—H2AA···O1^iii^	0.97	2.49	3.417 (5)	161
C6A—H6AA···O5A^iv^	0.93	2.60	3.354 (6)	139

Symmetry codes: (i) *x*, *y*−1, *z*; (ii) −*x*+1, *y*+1/2, −*z*; (iii) −*x*+1, *y*−1/2, −*z*; (iv) −*x*+2, *y*+1/2, −*z*+1.
